# Sequential HIPEC, Claudin18.2-targeted therapy, and CapeOx chemotherapy leading to resolution of peritoneal metastases and curative resection in gastric cancer: a case report and literature review

**DOI:** 10.3389/fimmu.2025.1641424

**Published:** 2025-11-18

**Authors:** Weidong Li, Xiaodong Huang, Xiaowen Han, Jiayi Zhang, Bin Ma, Zhenyu Yin, Yuhan Wang, Lei Gao, Jianming Shi, Hao Chen

**Affiliations:** 1 Lanzhou University Second Hospital, Lanzhou, China; 2 Department of Surgical Oncology, Lanzhou University Second Hospital, Lanzhou, China; 3 Key Laboratory of the Environmental Oncology of Gansu Province, Lanzhou University Second Hospital, Lanzhou, China

**Keywords:** gastric cancer, peritoneal metastasis, HIPEC, Claudin18.2, targeted therapy

## Abstract

Gastric cancer, a leading global cause of cancer-related mortality, is frequently diagnosed at advanced stages with peritoneal metastasis, significantly limiting curative options. Current strategies integrate systemic chemotherapy, hyperthermic intraperitoneal chemotherapy (HIPEC), and targeted therapies, yet outcomes remain suboptimal. Claudin18.2, a tight junction protein overexpressed in 27.4–52% of gastric cancers, has emerged as a novel therapeutic target, with recent Phase III trials demonstrating survival benefits for Claudin18.2-targeted monoclonal antibodies in HER2-negative advanced disease. However, the synergy of HIPEC with Claudin18.2-targeted therapies remains unexplored. This study presents a case of Claudin18.2-positive advanced gastric adenocarcinoma with peritoneal metastasis treated with a multimodal regimen: HIPEC, systemic CapeOx chemotherapy, and Claudin18.2-targeted therapy (ASKB589). Following 12 cycles, imaging and tumor markers (CA125, CEA) normalized, enabling curative gastrectomy. At 893 days post-diagnosis, the patient remains disease-free (PFS/DFS: 893/573 days) on adjuvant capecitabine. Treatment-related toxicities (predominantly Grade 1–3 hematologic and gastrointestinal events) were manageable. This case highlights Claudin18.2 as an actionable biomarker and proposes a “local-systemic” synergy model for peritoneal-metastatic gastric cancer. However, the absence of cytoreductive surgery and single-case limitations necessitate validation through randomized trials. Future research should prioritize biomarker-driven HIPEC protocols (e.g., nanoparticle-enhanced Claudin18.2-targeted delivery) and dynamic monitoring of Claudin18.2 expression during therapy.

## Introduction

Gastric cancer is a common malignant tumor with a poor prognosis, ranking as the fifth most prevalent cancer and the fifth leading cause of cancer-related mortality globally (1, 2). Surgery is currently regarded as the sole curative treatment (3). However, most patients are diagnosed at advanced stages, missing the optimal window for surgical intervention. Approximately 50% of advanced gastric cancer cases with distant metastasis involve peritoneal dissemination, which clinically manifests as hepatogenic pain, ascites, and other symptoms (4). The primary therapeutic strategies for advanced gastric cancer include comprehensive regimens combining neoadjuvant chemoradiotherapy, molecular targeted therapy, and immunotherapy (4). For patients with peritoneal metastasis, current approaches involve preoperative neoadjuvant intraperitoneal and systemic combination chemotherapy, hyperthermic HIPEC combined with cytoreductive surgery, and multimodal therapies integrating intraperitoneal, intravenous, and oral administration (5). Intraperitoneal perfusion therapy, which combines hyperthermia and chemotherapy, enhances tumor cell sensitivity to chemotherapeutic agents, increases local drug concentration, and reduces systemic adverse effects (6). As peritoneal metastasis represents a localized manifestation of systemic disease, systemic chemotherapy remains the cornerstone of treatment, with intraperitoneal chemotherapy serving as an adjunct (7). Claudin18.2, a highly selective tight junction protein, is normally expressed exclusively in differentiated gastric mucosal epithelial cells but is retained during malignant transformation. It is expressed in a significant proportion of primary gastric cancers and their metastases, emerging as a promising therapeutic target following HER2 (8). The reported positivity rates for Claudin18.2, defined by moderate-to-strong immunohistochemical (IHC) expression, vary between 27.4% and 52% across studies due to differences in IHC methodologies, positivity thresholds, and patient populations (9–13). This prevalence markedly exceeds that of HER2, positioning Claudin18.2 as a potential biomarker for targeted therapy, tumor diagnosis, treatment efficacy evaluation, and prognosis prediction. To date, no relevant domestic or international literature has reportedxon the combination of HIPEC with Claudin18.2-targeted therapy for Claudin18.2-positive gastric cancer patients with peritoneal metastasis. Herein, we present a case of HIPEC followed by Claudin18.2-targeted therapy combined with CapeOx chemotherapy, which achieved complete resolution of peritoneal metastases and enabled curative resection, accompanied by a literature review.

## Case presentation

**Figure 1:**
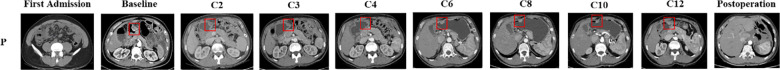
Abdominal CT imaging at admission and follow-up. (P: Primary lesion; First Admission: Initial presentation; Baseline: Diagnosis confirmation; C2–C12: CT scans at different treatment cycles; Postoperation: Post-total gastrectomy CT).

A 39-year-old male presented with a 10-day history of right lower quadrant abdominal pain and was admitted on December 6, 2022. He denied a history of hypertension, diabetes, or cardiovascular disease, as well as a family history of cancer. Eight months prior, the patient experienced right lower quadrant pain of unknown etiology and was diagnosed with acute appendicitis at a local hospital, which resolved after one week of antibiotic therapy. One month before admission, he underwent gastroscopy revealing gastric ulcer and chronic non-atrophic gastritis, treated symptomatically with acid suppression without further investigation. Ten days prior to admission, the pain recurred and failed conservative management. Abdominal non-contrast CT (from the diaphragmatic dome to the pelvic inlet) demonstrated a thickened appendix with intraluminal fecalith, adjacent mesenteric exudate, lymphadenopathy, and signs of peritonitis, suggestive of acute appendicitis. Tumor screening via contrast-enhanced CT was recommended (**Figure 1**). Diagnostic laparoscopy on December 8, 2022, revealed moderate ascites and widespread peritoneal and omental tumor nodules, consistent with metastatic malignancy (**Supplementary Figure S1**). Four HIPEC catheters were placed at the bilateral subcostal midclavicular lines and McBurney’s points. Postoperative pathology of the omental biopsy confirmed poorly differentiated adenocarcinoma (**Supplementary Figure S2**). IHC showed positivity for pan-CK and CK8/18, negativity for HER2, and a high proliferation index (Ki-67 ~80%). Ascitic cytology revealed severe nuclear atypia (**Supplementary Figure S2**). Subsequent gastroscopy identified a raised, ulcerated lesion on the greater curvature of the stomach (**Supplementary Figure S3**). Biopsy confirmed poorly differentiated adenocarcinoma with marked nuclear atypia, IHC positivity for CKpan and CK8/18, HER2 negativity (0), and a Ki-67 index of 80%. The final diagnosis was gastric adenocarcinoma (cT4aN2M1) with peritoneal metastasis. Karnofsky performance status (KPS) was 80, ECOG score 1, VAS score 2, and NRS score 2. IHC further revealed moderate Claudin18.2 expression (3+ [strong] in 10%, 2+ [moderate] in 30%, 1+ [weak] in 50%, and negative in 10%) and PD-L1 negativity (TPS <1%; CPS = 2) (**Supplementary Figure S2**). It fulfills the inclusion criteria pertaining to moderate-high Claudin18.2 expression levels for the ASKB589 Injection clinical trial. This testing was performed as an Laboratory Developed Test assay at the central laboratory of Medx Translation Medicine Research (Suzhou) Co., Ltd., using the Claudin 18.2 antibody reagent (immunohistochemistry) kit manufactured by Medx (Catalog No.: P054B01.a), with staining results interpreted based on a percentage threshold.

**Figure 2:**
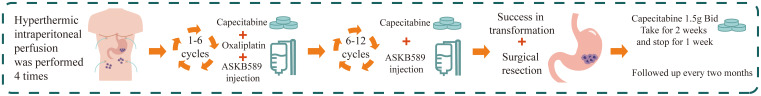
Treatment schema: HIPEC combined with CapeOx chemotherapy, Claudin18.2-targeted therapy, and curative gastrectomy.

**Figure 3:**
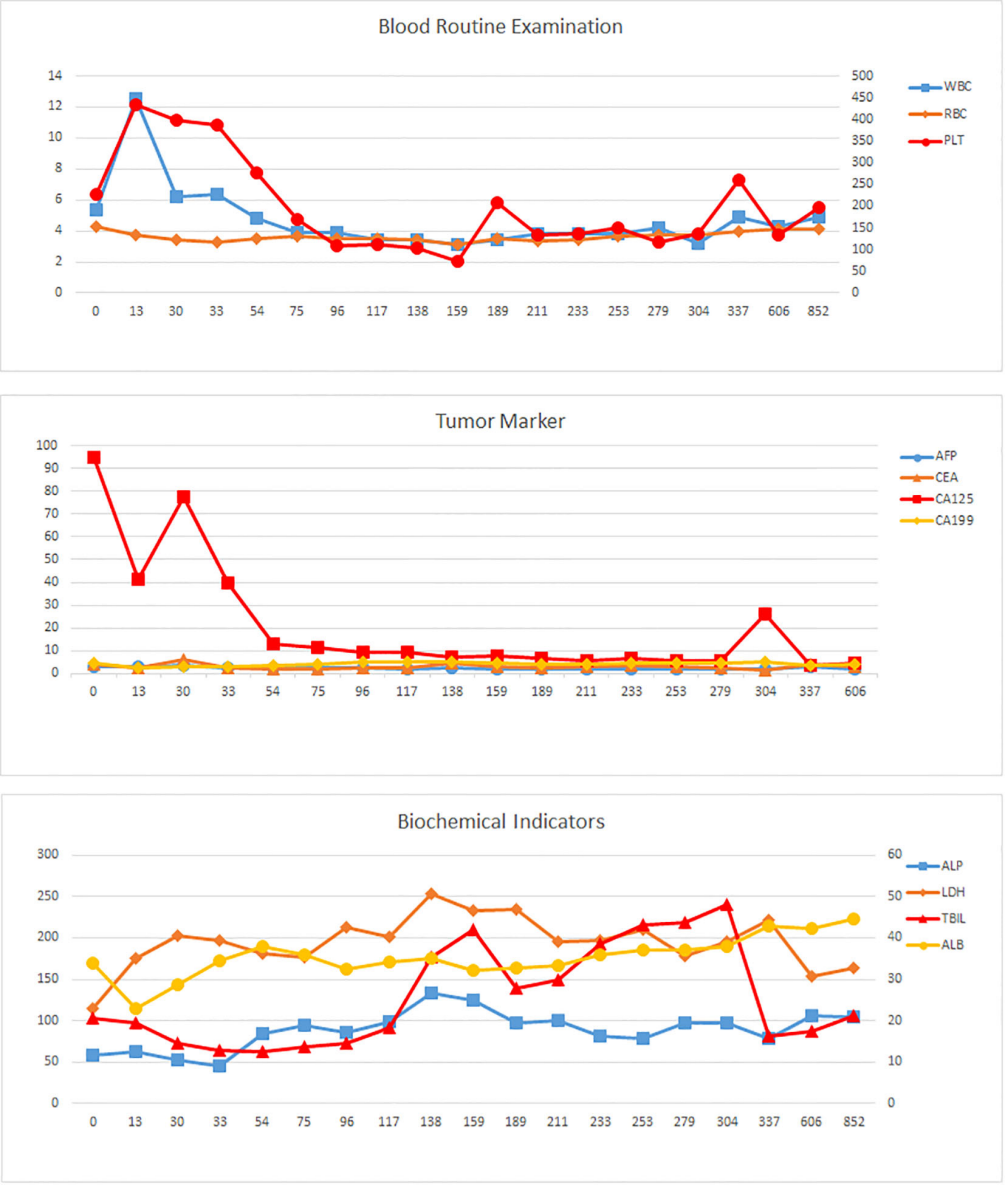
Laboratory parameter trends. Tumor markers (AFP, CEA, CA19-9, CA125); hematologic indices (PLT, WBC, RBC); biochemical profiles (TBIL, ALB, ALP, LDH). The horizontal axis represents the number of days since the diagnosis.

A multidisciplinary team recommended HIPEC combined with Claudin18.2-targeted therapy (ASKB589, 6 mg/kg every 3 weeks) and systemic chemotherapy. HIPEC involved 4500 mL perfusate at 43°C for 60 minutes (flow rate: 500 mL/min). Six cycles of CapeOx (oxaliplatin 130 mg/m² on Day 1; capecitabine 1000 mg/m² twice daily on Days 1–14, every 3 weeks) were administered, followed by six cycles of capecitabine monotherapy. Post-treatment CT and gastroscopy demonstrated partial response with reduced gastric wall thickening (**Figure 2**). Tumor markers (CA125, CEA) normalized by February 20, 2023. Transient hyperbilirubinemia and hypoalbuminemia resolved postoperatively (**Figure 3**). During the entire treatment course, the patient experienced a total of 41 adverse events, predominantly hematologic toxicities and gastrointestinal reactions. Hematologic toxicities included neutropenia (4 events, with 1 Grade 3 occurrence), thrombocytopenia (3 events), leukopenia (3 events), and anemia (1 event). Gastrointestinal reactions were most frequently observed as vomiting (11 Grade 1 events) and nausea (7 Grade 1 events). Other abnormalities included occult blood positivity in urine and stool (2 events each) and mild elevations in liver function indices (1 event each for bilirubin and aspartate aminotransferase [AST]). No Grade 4 events were reported, and only 1 Grade 3 neutropenia event occurred. All other events were Grade 1–2 and resolved with supportive care. Overall, the treatment demonstrated manageable safety, though close monitoring for myelosuppression remained warranted. (**Table 1**).

**Table 1:** Adverse events during treatment and grading.

Adverse Event	Any grade (n=41)	Grade 1	Grade 2	Grade 3	Grade 4
Any adverse event	41	32	4	1	0
Vomiting	11	11	–	–	–
Nausea	7	7	–	–	–
Abdominal pain	2	2	–	–	–
Gastric retention	1	1	–	–	–
Constipation	1	1	–	–	–
Positive urine occult blood	2	–	–	–	–
Positive fecal occult blood	2	–	–	–	–
Leukopenia	3	2	1	–	–
Neutropenia	4	1	2	1	–
Lymphopenia	1	1	–	–	–
Anemia	1	1	–	–	–
Thrombocytopenia	3	2	1	–	–
Hyperbilirubinemia	1	1	–	–	–
Elevated AST	2	2	–	–	–

Following tumor diagnosis on December 8, 2022, the patient underwent four cycles of HIPEC, 12 cycles of systemic therapy (6 cycles of CapeOx regimen [oxaliplatin + capecitabine] and 6 cycles of capecitabine monotherapy), and 12 cycles of Claudin18.2-targeted therapy. After completing 12 treatment cycles, successful conversion therapy enabled curative gastrectomy on October 24, 2023 (**Figure 2**). Postoperative histopathology revealed poorly differentiated gastric adenocarcinoma with treatment-related fibrotic and histiocytic reactions. According to NCCN guidelines, the tumor regression grade (TRG) was assessed as Grade 3, resulting in a final pathological stage of pT3N1M0. Omental specimens demonstrated foamy histiocyte proliferation, multinucleated giant cell reactions, and treatment-induced changes, with no residual carcinoma identified. Postoperative contrast-enhanced CT confirmed successful total gastrectomy with patent anastomosis and no significant wall thickening or abnormal enhancement at the anastomotic site. The patient has remained alive with adjuvant capecitabine maintenance therapy, achieving an overall survival (OS) and progression-free survival (PFS) of 893 days and a disease-free survival (DFS) of 573 days as of May 19, 2025.

## Discussion and conclusion

Gastric cancer, a malignancy with poor prognosis, is often diagnosed at advanced stages, leaving many patients ineligible for curative surgery. Peritoneal metastasis, a common complication in advanced gastric cancer, predominantly manifests as malignant ascites or intestinal obstruction (14, 15). Current therapeutic strategies for metastatic gastric cancer emphasize systemic pharmacotherapy, including chemotherapy, molecular-targeted agents, and immune checkpoint inhibitors (16). Clinically validated biomarkers for targeted therapy selection in advanced gastric/gastroesophageal junction cancer are limited to HER2 positivity, microsatellite instability (MSI) status, and PD-L1 expression (17). Emerging biomarkers under investigation include FGFR2 amplification/overexpression, MET amplification, Claudin18.2 (Claudin18.2) overexpression, and Epstein-Barr virus (EBV) association (18). HER2, expressed in 7.3%–20.2% of gastric cancers, remains a key therapeutic target, with trastuzumab combined with chemotherapy established as the first-line standard for HER2-positive disease (19, 20).

**Figure 4:**
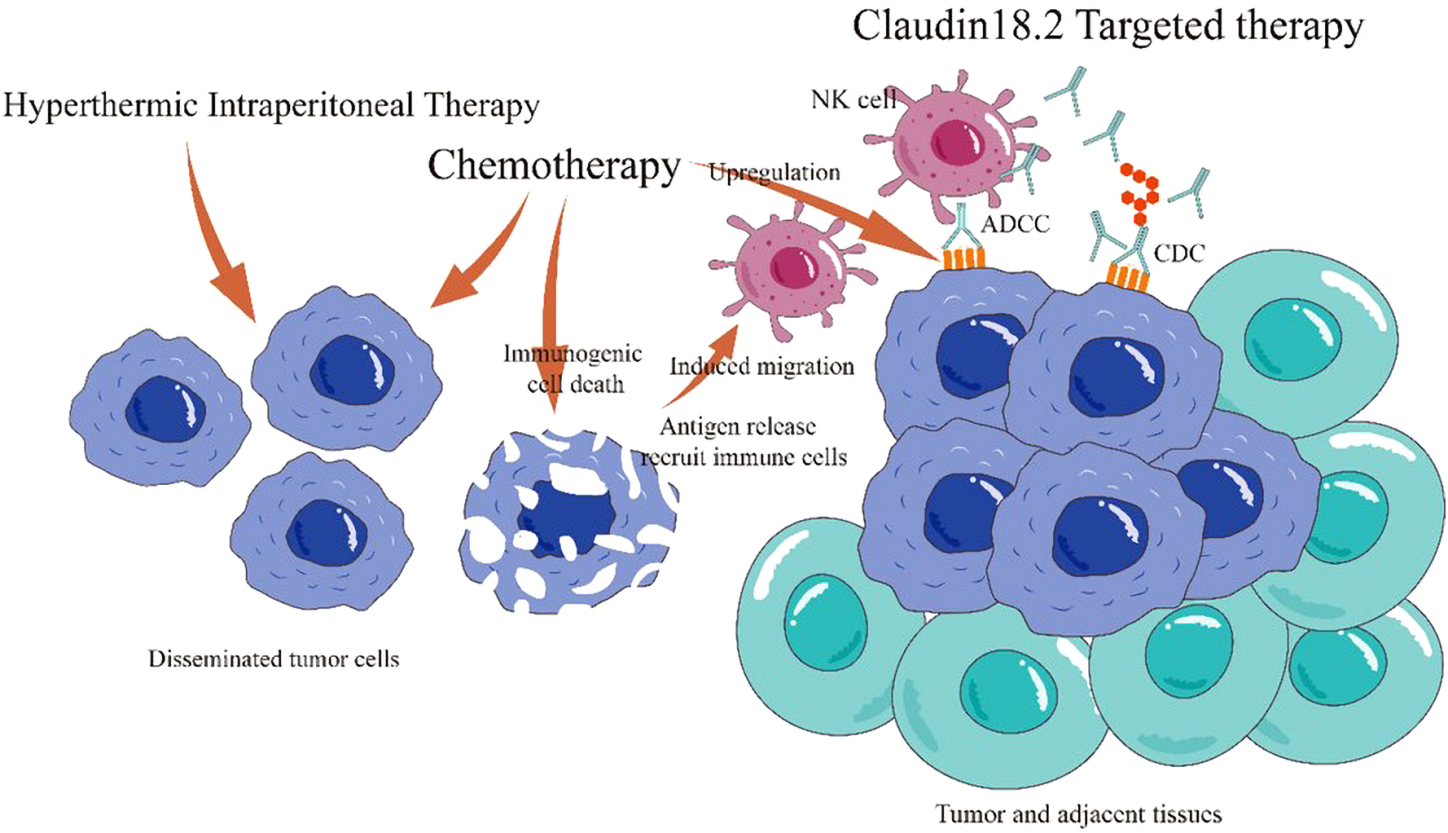
Mechanisms of combined HIPEC, chemotherapy, and Claudin18.2-targeted therapy. HIPEC eradicates free peritoneal tumor cells and prevents dissemination. Systemic chemotherapy targets primary and metastatic lesions. Claudin18.2-targeted therapy induces tumor cell death via antibody-dependent cellular cytotoxicity (ADCC) and complement-dependent cytotoxicity (CDC). Chemotherapy upregulates Claudin18.2 expression, enhancing targeted cytotoxicity, and promotes immunogenic cell death, improving the tumor immune microenvironment.

Claudin18.2, a tight junction protein overexpressed in 27.4%–52% of gastric cancers, has emerged as a promising biomarker and therapeutic target; While its expression varies across studies due to differences in immunohistochemical (IHC) methodologies and thresholds, its stability and selectivity support its potential role in diagnosis, treatment response assessment, and prognosis prediction (9–13, 21). Current Claudin18.2-targeted therapies under clinical evaluation include monoclonal antibodies (e.g., zolbetuximab), bispecific antibodies, CAR-T cells, and antibody-drug conjugates (ADCs). Phase III trials such as GLOW (NCT03653507) and SPOTLIGHT demonstrated significant improvements in progression-free survival (PFS) and overall survival (OS) with zolbetuximab combined with CAPOX or mFOLFOX6 versus chemotherapy alone in Claudin18.2-positive, HER2-negative advanced gastric/GEJ cancer (9, 22). Notably, the 2023 CSCO guidelines now recommend Claudin18.2 IHC testing (Category 2B, Level II evidence) (23). While systemic chemotherapy faces limitations in penetrating the blood-peritoneal barrier, HIPEC enhances local drug concentration and efficacy. In this case, HIPEC followed by CapeOx chemotherapy and claudin18.2-targeted therapy (ASKB589) achieved regression of peritoneal metastases and tumor downstaging, which enabled a curative resection. Potential synergistic mechanisms include HIPEC-mediated eradication of free peritoneal tumor cells, systemic chemotherapy targeting micrometastases, and Claudin18.2-targeted antibody-dependent cellular cytotoxicity (ADCC) and complement-dependent cytotoxicity (CDC) (13). Preclinical studies suggest chemotherapeutics like oxaliplatin may upregulate Claudin18.2 expression and enhance immunogenic cell death, further potentiating targeted therapy (24–26) (**Figure 4**).

This case has several inherent limitations, including the absence of cytoreductive surgery combined with HIPEC-chemotherapy, the lack of dynamic monitoring of CLDN18.2 expression during treatment, and—notably—the fact that targeted therapy was based solely on biopsy results from the primary lesion, without tissue sampling from peritoneal metastases. The latter point is particularly relevant in light of recent data published by Saito et al., which revealed that CLDN18.2 expression in peritoneal metastases is often significantly lower than in primary tumors (27). Therefore, the remarkable efficacy observed in this patient should be interpreted with caution, as it may reflect unique tumor biology rather than generalizable treatment effects. To address these gaps, future studies should incorporate both planned repeat biopsies to elucidate therapy-induced modulation of CLDN18.2 expression and synchronous CLDN18.2 analysis of paired primary and metastatic sites—especially peritoneal metastases—in order to clarify heterogeneity patterns, determine the optimal biopsy site for treatment guidance, and ultimately optimize therapeutic sequencing to overcome resistance. Larger randomized trials will be essential to validate this multimodal approach.

## Data Availability

The raw data supporting the conclusions of this article will be made available by the authors, without undue reservation.

## Supplementary material

Supplementary Figure 1 Laparoscopic findings showing widespread peritoneal and omental tumor nodules, indicative of malignant peritoneal metastasis.

Supplementary Figure 2 Pathological examination results. **(A)** Omental biopsy: Poorly differentiated adenocarcinoma within fibrous and lymphoid tissue. **(B)** Ascitic fluid cytology: Severely dysplastic cells. **(C)** Gastric biopsy: Loss of glandular architecture with fibrotic stroma and diffusely arranged atypical epithelial cells exhibiting hyperchromatic nuclei. Immunohistochemistry revealed Claudin18.2 tumor staining intensity: 3+ (10%), 2+ (30%), 1+ (50%), and negative (10%); PD-L1 negativity (TPS <1%, CPS = 2).

Supplementary Figure 3 Endoscopic progression of the gastric lesion. **(A)** Baseline diagnosis; **(B)** After 4 cycles of HIPEC; **(C–H)** Sequential endoscopic findings post-Cycle 2 (C2) through Cycle 12 (C12).
