# Supplementary figures and images for: Sequential HIPEC, Claudin18.2-targeted therapy, and CapeOx chemotherapy leading to resolution of peritoneal metastases and curative resection in gastric cancer: a case report and literature review
Source: Front Immunol. 2025 Nov 18;16:1641424. doi: 10.3389/fimmu.2025.1641424 (PMC12669137; doi:10.3389/fimmu.2025.1641424)

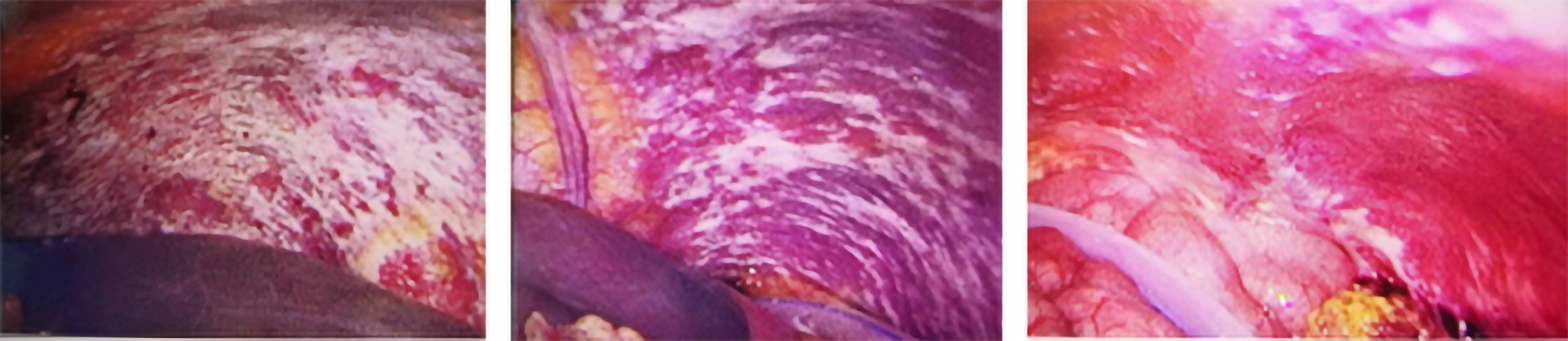

Supplement: Supplementary Figure 1 — Laparoscopic findings showing widespread peritoneal and omental tumor nodules, indicative of malignant peritoneal metastasis. [file Image1.jpg]

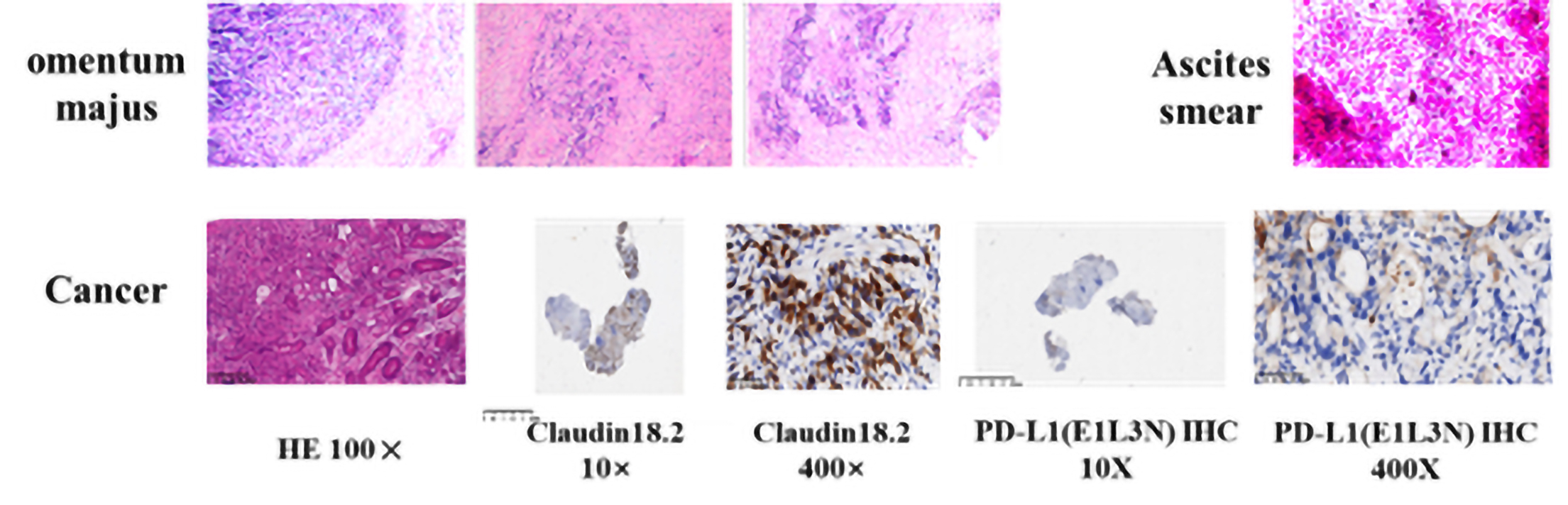

Supplement: Supplementary Figure 2 — Pathological examination results. (A) Omental biopsy: Poorly differentiated adenocarcinoma within fibrous and lymphoid tissue. (B) Ascitic fluid cytology: Severely dysplastic cells. (C) Gastric biopsy: Loss of glandular architecture with fibrotic stroma and diffusely arranged atypical epithelial cells exhibiting hyperchromatic nuclei. Immunohistochemistry revealed Claudin18.2 tumor staining intensity: 3+ (10%), 2+ (30%), 1+ (50%), and negative (10%); PD-L1 negativity (TPS <1%, CPS = 2). [file Image2.jpg]

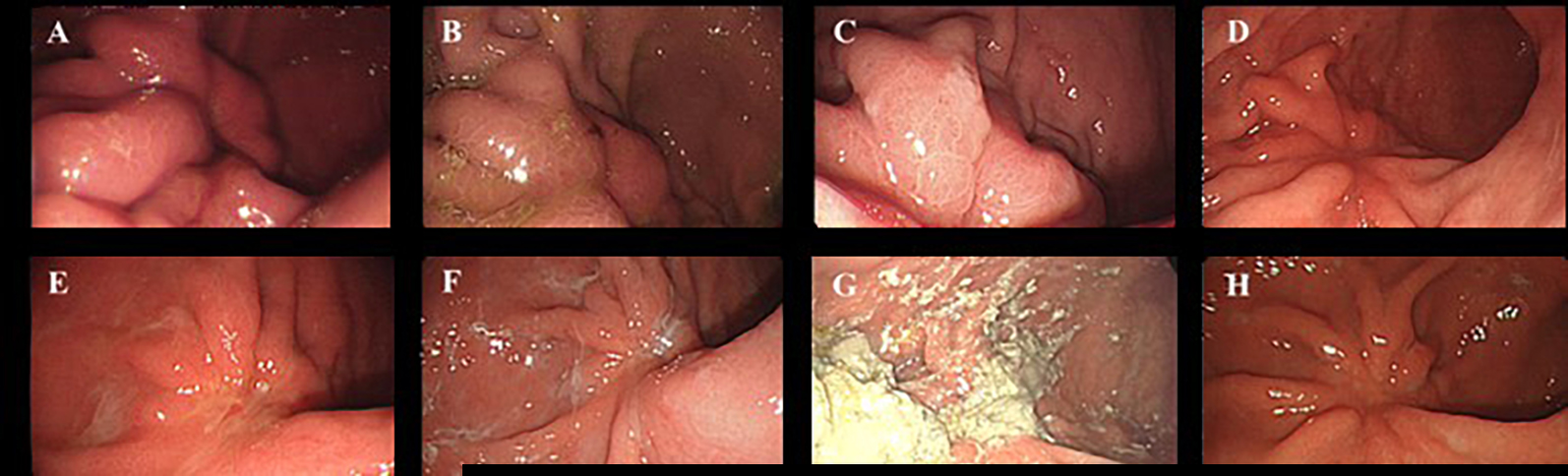

Supplement: Supplementary Figure 3 — Endoscopic progression of the gastric lesion. (A) Baseline diagnosis; (B) After 4 cycles of HIPEC; (C–H) Sequential endoscopic findings post-Cycle 2 (C2) through Cycle 12 (C12). [file Image3.jpg]
